# Quality of Life of Patients With Stress Urinary Incontinence Before and After Transobturator Tape Surgery: An Observational Study

**DOI:** 10.7759/cureus.76092

**Published:** 2024-12-20

**Authors:** Indrani Dutta, Baidyanath Pathak

**Affiliations:** 1 Obstetrics and Gynecology, Manipal Tata Medical College, Jamshedpur, IND

**Keywords:** incontinence impact questionnaire, incontinence quality of life questionnaire, long-term outcomes, pelvic floor surgery, quality of life, stress urinary incontinence, transobturator tape procedure

## Abstract

Introduction

Stress urinary incontinence (SUI) is the involuntary leakage of urine during physical activities such as coughing, sneezing, or exercising. It can significantly affect the quality of life (QOL) of many women by causing embarrassment, reducing confidence, and limiting social or physical activities. SUI results from increased intra-abdominal pressure due to physical exertion. The transobturator tape (TOT) procedure is a common surgical intervention for SUI. This study aimed to evaluate the impact of TOT on the QOL of patients with SUI using validated instruments.

Methods

This prospective observational study included 59 women with moderate to severe SUI treated at a tertiary care hospital in Eastern India from December 2022 to April 2024. Patients underwent the TOT procedure, and QOL was assessed preoperatively and at six- and 12-month follow-ups using the Incontinence Impact Questionnaire (IIQ-7) and the Incontinence Quality of Life (I-QOL) questionnaire. Statistical analysis was conducted using SPSS version 16.0 (SPSS Inc., Chicago, Ill), with Wilcoxon signed-rank tests applied to evaluate pre-post differences.

Results

Participants experienced significant improvements in QOL across all domains of both questionnaires. The mean IIQ-7 score decreased from 69.09 preoperatively to 11.22 at 12 months (p<0.001), indicating a reduced impact of SUI on daily activities. I-QOL scores also improved, with the "Avoidance and Limiting Behavior" domain increasing from 17.48 preoperatively to 64.94 at 12 months (p<0.001). Similar improvements were observed in the "Psychosocial Impacts" and "Social Embarrassment" domains. These results reflect enhanced social, emotional, and physical well-being in the long term following this surgical procedure.

Conclusion

The TOT procedure significantly improves the QOL of women with SUI. Patients reported reduced physical limitations, social embarrassment, and psychological distress postoperatively. These findings emphasize the importance of surgical interventions such as TOT in alleviating the multifaceted burden of SUI. Further research with larger samples is recommended to confirm the long-term benefits.

## Introduction

Urinary incontinence (UI) remains an under-recognized medical concern. The International Continence Society defines UI as "any involuntary loss of urine," categorizing it into stress urinary incontinence (SUI), urge urinary incontinence (UUI), and mixed urinary incontinence (MUI) [1,2]. According to the World Health Organization, UI affects approximately 5% of the global population [3], with a prevalence of 30%-60% among perimenopausal and postmenopausal women [4].

SUI is the most common form of UI, accounting for about 50% of cases in women [5]. It is characterized by involuntary urine leakage caused by sudden increases in intra-abdominal pressure during activities such as coughing, sneezing, laughing, running, or other physical exertions [6]. Several factors contribute to the development of SUI, including aging, instrumental vaginal delivery, heavy lifting, reduced tissue strength, nerve damage, hypoestrogenism, a high body mass index, and weakened pelvic floor muscles [7].

SUI significantly impacts patients' quality of life (QOL), negatively affecting both physical and mental well-being [8]. Many individuals with urinary incontinence withdraw from social interactions due to embarrassment or fear of being socially ostracized. This reluctance often prevents them from seeking timely medical help or discussing their symptoms.

A commonly used surgical treatment for SUI involves mid-urethral slings, performed using either a retropubic or transobturator approach. Studies suggest that the transobturator tape (TOT) procedure results in greater improvements in QOL compared to the tension-free vaginal tape (TVT) procedure [9].

Despite its widespread use, research evaluating the long-term effects of the TOT procedure on QOL is limited [10,11], particularly in the Indian patient population [12]. Therefore, this study aims to assess the QOL of patients with stress incontinence before and after undergoing surgery using the TOT method.

## Materials and methods

The present prospective observational study was conducted in the Department of Gynecology and Obstetrics in a tertiary care hospital in Eastern India from December 2022 to April 2024 after approval from the Institutional Ethics Committee (approval number: TMH/AC/IEC/NOV/062/2022). The study has been reported in line with the Strengthening the Reporting of Observational Studies in Epidemiology (STROBE) guidelines.

Study participants

The study sample was taken from the outpatient department (OPD), with participants being women presenting with SUI, giving consent, being surgically fit for the TOT procedure, and willing to come for follow-up at six and 12 months after surgery. The sample size for the study was calculated using the standard formula for prevalence study, given as n = Z_{1-\alpha/2}^{2}\text{ }p\text{ }(1-p)/d^{2}. In the study conducted by Ajith et al. (2019) in India, the prevalence of SUI among postmenopausal women was reported to be 13.9% [13]. Taking prevalence in our study setting to be 13.9% and considering a 95% confidence limit and 10% absolute error, the minimum calculated sample size was 46. Owing to the long follow-up period, allowance was taken assuming a 20% loss to follow-up. Therefore, a total of 59 patients were included in the study.

Study procedure

All patients presenting to the Gynecology OPD with complaints of SUI were evaluated. They were asked to keep a record of incontinence and to take a one-hour pad test where used pads were weighed before and after one-hour usage. Pads were kept at the OPD for distribution and were provided to the patients. The severity of SUI was classified as follows, as defined by Ferreira and Bø [14]: mild SUI, light urine loss quantity, maximally until 10 g/24 hour; moderate SUI, moderate urine loss, quantity of 11-50 g/24 hour; and severe SUI, heavy urine loss, quantity over 50 g/24 hour.

In the present study, only patients with moderate to severe SUI who were declared surgically fit under pre-anesthetic evaluation and gave consent for surgery were considered for the TOT procedure. Those patients on "hormone replacement therapy, with overactive bladders, mixed urinary incontinence, with urinary tract fistulas, with congenital or acquired defects of the urethra or the bladder, with urinary tract infections, and taking medication leading to an overactive bladder" were excluded from the study [11].

The operative steps for the TOT procedure were as follows. The patient was placed in a lithotomy position with stirrups. Vasopressin infiltration was done suburethral position radially, and a suburethral single vaginal incision was given. The pathway for device placement was created by dissecting vaginal flaps suburethrally on both sides of the midline. Insertion of the obturator needle (right side of the patient) was done from a point that was present at the intersection of a vertical line drawn from the insertion of the adductor longus and a horizontal line drawn from the tip of the clitoris. The needle was inserted at this point and gently traversed negotiating the obturator space to be taken out from suburethral space. At this point, TOT mesh was threaded over the obturator needle, and the needle along with the mesh was taken out through its insertion point. The needle was freed from the mesh, and the mesh was cut to a length close to the skin insertion point. The same procedure was repeated for the left side of the patient, and the mesh was placed at the suburethral position in a semilunar manner. The flat position of the tape was checked, and the correct tension of the mesh was obtained by keeping a mayo scissor beneath the suture suburethrally, which was subsequently removed. Both lateral arms of the mesh emerging from the skin were sutured with vicryl number 1 with the overlying skin to be removed after 10 days. Urinary drainage was checked to exclude stenosis, and vaginal mucosa and skin were sutured subsequently.

Any patient presenting with pelvic organ prolapse and chosen for either vaginal hysterectomy with pelvic floor repair or site-specific repair was subsequently operated on as decided before surgery.

Outcome measures

Patients enrolled in the study completed the Incontinence Impact Questionnaire (IIQ-7) and the Incontinence Quality of Life (I-QOL) questionnaire. These assessments were conducted at three time points: upon admission before undergoing the TOT procedure and at follow-up visits six months and 12 months post-procedure.

The IIQ-7 is a concise version of the original Incontinence Impact Questionnaire (IIQ), designed to evaluate the impact of urinary incontinence on a patient's QOL. It consists of seven questions addressing areas such as household activities, physical activities, entertainment, travel, leisure, and mental health. Each question is scored on a scale of 0 to 3, where 0 indicates not at all, 1 indicates a little, 2 indicates medium, and 3 indicates very. The mean score of the responses is calculated and converted to a scale of 0-100 by the unitary method [15]. Higher scores correspond to worse symptoms and a lower quality of life [16].

The I-QOL is a validated quality of life tool specifically for patients with urinary incontinence. It comprises 22 questions divided into three domains: Avoidance and Limiting Behavior, Psychosocial Impact, and Social Embarrassment. Each question is rated on a five-point scale: 1 = extremely, 2 = quite a bit, 3 = moderately, 4 = a little, and 5 = not at all [17]. The domain and total scores are calculated by summing item responses and transforming the results into a 100-point scale, where 0 indicates the most severe problems and 100 represents no issues [18].

Data entry and statistical analysis

Data were recorded using a predesigned and pretested proforma. The collected data were entered into Microsoft Excel 2016 (Microsoft Corp., Redmond, WA) and analyzed using SPSS version 16.0 (SPSS Inc., Chicago, Ill), with findings presented in tables, graphs, and diagrams. Continuous variables were expressed as mean ± standard deviation (SD), and categorical variables were represented as relative frequencies and percentages. A comparison of study outcomes was done for categorical variables using the Chi-square test or Fisher's exact test (as applicable). Pre-post differences in the IIQ-7 scores and I-QOL total and domain scores were tested using the Wilcoxon signed-rank test for paired samples. A p-value < 0.05 was considered statistically significant.

## Results

A final analysis was done on data collected from 59 participants. The mean (SD) age of the patients was 60.28 (4.6) years and ranged from 51 to 77 years. Our analysis revealed a significant improvement in the domain scores of the IIQ-7 from the preoperative period to six and 12 months postoperatively. The mean score decreased significantly from 69.09 (SD=11.02) preoperatively to 51.90 (SD=9.55) at six months (p<0.001) and further to 11.22 (SD=6.95) at 12 months (p<0.001). Similarly, the median scores showed a consistent decline, from 66.67 preoperatively to 52.38 at six months and 9.52 at 12 months (Table 1).

**Table 1:** Domain scores of the IIQ-7 preoperatively and at six and 12 months postoperatively (N=59) ^a^Significant at p<0.001 (calculated using the Wilcoxon signed-rank test between observations taken preoperatively and at six months postoperatively) ^b^Significant at p<0.001 (calculated using the Wilcoxon signed-rank test between observations taken preoperatively and at 12 months postoperatively) IIQ-7: Incontinence Impact Questionnaire, SD: standard deviation

Parameters	Preoperatively	At 6 months postoperatively	At 12 months postoperatively
Mean (SD)	69.09 (11.02)	51.90 (9.55)^a^	11.22 (6.95)^b^
Median (range)	66.67 (38.10-90.48)	52.38 (33.33-71.43)	9.52 (0-33.33)

On the item-wise evaluation of responses to the IIQ-7, a marked improvement was noted from the preoperative period to six and 12 months postoperatively. Across all domains, there is a significant reduction in the proportion of participants reporting "moderate" and "great" impacts of their ailments, with a corresponding increase in those reporting "slight" or "no" impact (Appendices).

Table 2 highlights the significant postoperative improvements across all domains of the I-QOL questionnaire. In the "Avoidance and Limiting Behavior" domain, mean (SD) scores increased from 17.48 (7.96) preoperatively to 45.50 (6.90) at six months (p<0.001) and further to 64.94 (9.08) at 12 months. A similar trend was observed in the "Psychosocial Impacts" domain, with mean scores rising from 16.95 (7.36) preoperatively to 46.75 (6.08) at six months (p<0.001) and to 64.36 (12.94) at 12 months. The "Social Embarrassment" domain also showed marked improvements, with mean scores increasing from 15.76 (8.94) preoperatively to 47.20 (8.27) at six months and to 66.27 (16.65) at 12 months. These results indicate a significant enhancement in the patients' quality of life across all domains, with sustained benefits evident at the 12-month follow-up.

**Table 2:** Domain scores of the I-QOL questionnaire preoperatively and at six and 12 months postoperatively (N=59) ^a^Significant at p<0.001 (calculated using the Wilcoxon signed-rank test between observations taken preoperatively and at six months postoperatively) ^b^Significant at p<0.001 (calculated using the Wilcoxon signed-rank test between observations taken preoperatively and at 12 months postoperatively) I-QOL: Incontinence Quality of Life, SD: standard deviation

Domains	Parameters	Preoperatively	At 6 months postoperatively	At 12 months postoperatively
Avoidance and Limiting Behavior	Mean (SD)	17.48 (7.96)	45.50 (6.90)^a^	64.94 (9.08)
	Median (range)	18.75 (0-31.25)	46.88 (28.13-59.38)	65.63 (40.63-84.38)
Psychosocial Impacts	Mean (SD)	16.95 (7.36)	46.75 (6.08)^a^	64.36 (12.94)^b^
	Median (range)	16.67 (3.78-30.56)	47.22 (27.78-58.33)	63.89 (38.89-91.67)
Social Embarrassment	Mean (SD)	15.76 (8.94)	47.20 (8.27)	66.27 (16.65)
	Median (range)	15.00 (0-35)	8.27 (25-60)	70 (30-95)
Overall	Mean (SD)	16.87 (6.86)	46.40 (6.30)	65.00 (10.34)
	Median (range)	18.18 (30.68-57.95)	46.59 (30.68-57.95)	63.64 (40.91-88.64)

Domain-specific item-wise assessment of responses also showed significant improvements in the quality of life for participants concerning avoidance behaviors, psychological impacts, and social embarrassment related to urinary incontinence, from preoperative evaluations to six-month and 12-month postoperative follow-ups. The avoidance behaviors, such as concerns about reaching the toilet on time, coughing/sneezing, or traveling, showed a progressive shift from higher-impact responses (scores of 1 and 2) to lower-impact responses (scores of 3, 4, and 5) (Appendices). These changes, consistent across other items, indicate increased confidence and reduced limitations in daily activities (p<0.001).

Similarly, the psychological relief experienced by participants is shown in the Appendices. The proportion of individuals reporting severe depressive feelings or frustration (responses 1 and 2) dropped dramatically, with no participants in these categories by 12 months for several items.

Parallel improvements in social embarrassment metrics with fears such as being humiliated or uncontrolled leakage drastically reduced as highlighted in the Appendices. By 12 months, a significant portion of participants reported negligible embarrassment, with a shift toward scores of 3 to 5 (p<0.001).

## Discussion

The findings from this study highlight the significant improvement in QOL among patients with SUI following the TOT procedure. The results from the IIQ-7 revealed a marked reduction in the impact of SUI on daily activities, physical recreation, and social engagement. Before the surgery, many patients reported moderate to severe limitations in household chores, physical activities, and traveling, with no patients reporting no effect at all. However, by 12 months postoperatively, a substantial proportion (32.2% to 79.7% depending on the domain) reported that these ailments no longer affected their lives. This underscores the significant alleviation of symptoms and restoration of functionality following the TOT procedure.

Similarly, participation in social activities and emotional well-being showed remarkable improvement. Social embarrassment and emotional health scores revealed a shift from moderate to severe preoperative limitations to mild or no limitations postoperatively. These improvements are crucial as they reflect not only physical recovery but also enhanced social and psychological reintegration.

The I-QOL questionnaire provided further insight into psychological and emotional impacts. Before surgery, patients expressed feelings of depression, helplessness, and frustration due to their condition, with these symptoms affecting their confidence and social engagement. Postoperatively, a consistent decline in these adverse impacts was observed, with most patients reporting little to no psychological burden by the 12-month follow-up. The shift in responses from "greatly" or "moderately" affected to "slightly" or "not at all" indicates a profound improvement in emotional resilience and psychological health.

Quantitative analysis of the domain scores demonstrated significant and sustained improvements in all domains of the I-QOL questionnaire and IIQ-7 over the study period, reflecting an overall improvement in QOL.

Zalewski et al. also reported significant improvements in patients' QOL after undergoing the TOT procedure, using similar standardized QOL assessment tools [11]. Similarly, Kołodyńska et al. employed the World Health Organization Quality of Life (WHOQOL-BREF.FL) questionnaire to evaluate QOL in physically active and inactive elderly women before and after the TOT procedure, with consistently positive outcomes [10]. In another study, Jacob et al. used the two-part King's Health Questionnaire to assess QOL following the TOT method. Their findings confirmed that the procedure is both safe and effective for treating stress urinary incontinence (SUI), resulting in substantial enhancements in patients' QOL [12].

Orhan et al. observed that patients experiencing severe stress urinary incontinence reported a more pronounced decline in quality of life compared to those with mild symptoms [19]. Similarly, Bushnell et al. emphasized that urinary incontinence is not merely a physical health issue but also has significant social, emotional, and physical repercussions [20]. Many women with urinary incontinence limit their physical and social activities due to fear of leakage and are more likely to report sexual difficulties. These challenges, however, can improve with effective treatment, as demonstrated by the findings of this study [21].

The mental health effects of urinary incontinence were explored by Goldacre et al., who found that women with the condition are at a higher risk for depression and self-harm. Severe incontinence symptoms were associated with a threefold increase in severe depression compared to mild cases [22]. Irwin et al. in their study found that patients with incontinence experienced psychological discomfort and poor quality of life [23].

The drawback of our study is a small sample size, due to the long follow-up period, which limits the generalizability of the findings to broader demographic groups. On the other hand, the study evaluates the different aspects of QOL using condition-specific validated and standardized instruments, ensuring the reliability and comparability of the findings. Also, the long follow-up period allows us to develop an insight into the long-term impact of this surgical procedure on the quality of life of women suffering from SUI.

## Conclusions

The findings of the present study align with previous research emphasizing the TOT procedure as an effective and minimally invasive intervention for SUI. The sustained improvements in the QOL observed in this study reinforce the long-term efficacy and reliability of this approach. Furthermore, the significant psychological benefits underscore the importance of addressing the holistic needs of patients, including mental and emotional health, when treating SUI.

In conclusion, the TOT procedure not only provides relief from the physical symptoms of SUI but also significantly enhances patients' emotional well-being, social functioning, and overall QOL. This comprehensive improvement highlights the procedure's role as a transformative intervention for patients burdened by SUI. Further studies with larger cohorts would provide additional insights into the long-term outcomes and potential areas for optimizing patient care.

## Appendices

Table 3 shows the distribution according to responses to the IIQ-7 preoperatively and at six and 12 months postoperatively.

**Table 3:** Distribution according to responses to IIQ-7 preoperatively and at six and 12 months postoperatively (N=59) Values are presented as frequency (%). ^a^p-value was calculated using the Chi-square test between observations taken preoperatively and at six months postoperatively. ^b^p-value was calculated using the Chi-square test between observations taken preoperatively and at 12 months postoperatively. IIQ-7: Incontinence Impact Questionnaire

Items	Response	Preoperatively	At 6 months postoperatively	p-value^a^	At 12 months postoperatively	p-value^b^
1. How much do the ailments affect your ability to do household chores (cooking, housecleaning, laundry)?	Not at all	0 (0.0)	0 (0.0)	<0.001	19 (32.2)	<0.001
	Slightly	11 (18.6)	24 (40.7)		40 (67.8)	
	Moderately	29 (49.2)	30 (50.8)		0 (0.0)	
	Greatly	19 (32.2)	5 (8.5)		0 (0.0)	
2. How much do the ailments affect your physical recreation such as walking, swimming, or other exercise?	Not at all	0 (0.0)	0 (0.0)	<0.001	41 (69.5)	<0.001
	Slightly	19 (32.2)	30 (50.8)		14 (23.7)	
	Moderately	10 (16.9)	18 (30.5)		4 (6.8)	
	Greatly	30 (50.8)	11 (18.6)		0 (0.0)	
3. How much do the ailments affect your entertaining activities (movies, concerts, etc.)?	Not at all	0 (0.0)	0 (0.0)	<0.001	47 (79.7)	<0.001
	Slightly	25 (42.4)	40 (67.8)		11(18.6)	
	Moderately	13 (22.0)	14 (23.7)		1 (1.7)	
	Greatly	21 (35.6)	5 (8.5)		0 (0.0)	
4. How much do the ailments affect your ability to travel by car or bus more than 30 minutes from home?	Not at all	0 (0.0)	0 (0.0)	<0.001	44 (74.6)	<0.001
	Slightly	17 (28.8)	32 (54.2)		12 (20.3)	
	Moderately	24 (40.7)	20 (33.9)		3 (5.1)	
	Greatly	18 (30.5)	7 (11.9)		0 (0.0)	
5. Do the ailments affect your participation in social activities outside your home?	Not at all	0 (0.0)	0 (0.0)	<0.001	47 (79.7)	<0.001
	Slightly	21 (35.6)	35 (59.3)		8 (13.6)	
	Moderately	14 (23.7)	15 (25.4)		4 (6.8)	
	Greatly	24 (40.7)	9 (15.3)		0 (0.0)	
6. How much do the ailments affect your emotional health (nervousness, depression, etc.)?	Not at all	0 (0.0)	0 (0.0)	<0.001	46 (78.0)	<0.001
	Slightly	17 (28.8)	38 (64.4)		12 (20.3)	
	Moderately	19 (32.2)	17 (28.8)		1 (1.7)	
	Greatly	23 (39.0)	4 (6.8)		0 (0.0)	
7. How much do the ailments affect your feelings of frustration?	Not at all	0 (0.0)	0 (0.0)	<0.001	46 (78.0)	<0.001
	Slightly	15 (25.4)	32 (54.2)		10 (16.9)	
	Moderately	24 (40.7)	20 (33.9)		3 (5.1)	
	Greatly	20 (33.9)	7 (11.9)		0 (0.0)	

Table 4 shows the distribution according to avoidance and limiting behavior responses to the I-QOL questionnaire preoperatively and at six and 12 months postoperatively.

**Table 4:** Distribution according to avoidance and limiting behavior responses to I-QOL questionnaire preoperatively and at six and 12 months postoperatively (N=59) Values are presented as frequency (%). ^a^p-value was calculated using the Chi-square test between observations taken preoperatively and at six months postoperatively. ^b^p-value was calculated using the Chi-square test between observations taken preoperatively and at 12 months postoperatively. I-QOL: Incontinence Quality of Life

Items	Response	Preoperatively	At 6 months postoperatively	p-value^a^	At 12 months postoperatively	p-value^b^
1. I’m worried that I won’t be able to get to the toilet on time.	1	15 (25.4)	0 (0.0)	<0.001	0 (0.0)	<0.001
	2	32 (54.2)	13 (22.0)		5 (8.5)	
	3	12 (20.3)	36 (61.0)		24 (40.7)	
	4	0 (0.0)	10 (16.9)		27 (45.8)	
	5	0 (0.0)	0 (0.0)		3 (5.1)	
2. I am afraid of coughing/sneezing due to urinary incontinence.	1	28 (47.5)	0 (0.0)	<0.001	0 (0.0)	<0.001
	2	31 (52.5)	21 (35.6)		3 (5.1)	
	3	0 (0.0)	38 (64.4)		22 (37.3)	
	4	0 (0.0)	0 (0.0)		24 (40.7)	
	5	0 (0.0)	0 (0.0)		10 (16.9)	
3. I need to control myself when I’m getting up from a sitting position.	1	19 (32.2)	0 (0.0)	<0.001	0 (0.0)	<0.001
	2	32 (54.2)	18 (30.5)		11 (18.6)	
	3	8 (13.6)	38 (64.4)		19 (32.2)	
	4	0 (0.0)	3 (5.1)		22 (37.3)	
	5	0 (0.0)	0 (0.0)		7 (11.9)	
4. I worry about where the toilets are in a new place.	1	26 (44.1)	0 (0.0)	<0.001	0 (0.0)	<0.001
	2	33 (55.9)	17 (28.8)		1 (1.7)	
	3	0 (0.0)	34 (57.6)		25 (42.4)	
	4	0 (0.0)	8 (13.6)		25 (42.4)	
	5	0 (0.0)	0 (0.0)		8 (13.6)	
5. It is important for me to be able to use the toilet frequently.	1	26 (44.1)	0 (0.0)	<0.001	0 (0.0)	<0.001
	2	31 (52.5)	11 (18.6)		2 (3.4)	
	3	2 (3.4)	37 (62.7)		24 (40.7)	
	4	0 (0.0)	11 (18.6)		25 (42.4)	
	5	0 (0.0)	0 (0.0)		8 (13.6)	
6. It is important for me to plan every detail in advance because of urinary incontinence.	1	25 (42.4)	0 (0.0)	<0.001	0 (0.0)	<0.001
	2	33 (55.9)	22 (37.3)		4 (6.8)	
	3	1 (1.7)	37 (62.7)		26 (44.1)	
	4	0 (0.0)	0 (0.0)		26 (44.1)	
	5	0 (0.0)	0 (0.0)		3 (5.1)	
7. I have difficulty resting properly at night because of urinary incontinence.	1	18 (30.5)	0 (0.0)	<0.001	0 (0.0)	<0.001
	2	32 (54.2)	15 (25.4)		3 (5.1)	
	3	9 (15.3)	34 (57.6)		20 (33.9)	
	4	0 (0.0)	10 (16.9)		26 (44.1)	
	5	0 (0.0)	0 (0.0)		10 (16.9)	
8. I have to watch how much fluid I drink due to urinary incontinence.	1	23 (39.0)	0 (0.0)	<0.001	0 (0.0)	<0.001
	2	30 (50.8)	18 (30.5)		2 (3.4)	
	3	6 (10.2)	33 (55.9)		25 (42.4)	
	4	0 (0.0)	8 (13.6)		24 (40.7)	
	5	0 (0.0)	0 (0.0)		8 (13.6)	

Table 5 presents the distribution according to psychological impact responses to the I-QOL questionnaire preoperatively and at six and 12 months postoperatively.

**Table 5:** Distribution according to psychological impact responses to I-QOL questionnaire preoperatively and at six and 12 months postoperatively (N=59) Values are presented as frequency (%). ^a^p-value was calculated using the Chi-square test between observations taken preoperatively and at six months postoperatively. ^b^p-value was calculated using the Chi-square test between observations taken preoperatively and at 12 months postoperatively. I-QOL: Incontinence Quality of Life

Items	Response	Preoperatively	At 6 months postoperatively	p-value^a^	At 12 months postoperatively	p-value^b^
1. I feel depressed because of urinary incontinence.	1	23 (39.0)	0 (0.0)	<0.001	0 (0.0)	<0.001
	2	32 (54.2)	18 (30.5)		3 (5.1)	
	3	4 (6.8)	38 (64.4)		22 (37.3)	
	4	0 (0.0)	3 (5.1)		24 (40.7)	
	5	0 (0.0)	0 (0.0)		10 (16.9)	
2. I don’t feel comfortable enough to be out of the house for a long time because of urinary incontinence.	1	23 (39.0)	0 (0.0)	<0.001	0 (0.0)	<0.001
	2	30 (50.8)	11 (18.6)		11 (18.6)	
	3	6 (10.2)	37 (62.7)		19 (32.2)	
	4	0 (0.0)	11 (18.6)		22 (37.3)	
	5	0 (0.0)	0 (0.0)		7 (11.9)	
3. I feel frustrated because urinary incontinence restricts me from doing what I like.	1	27 (45.8)	0 (0.0)	<0.001	0 (0.0)	<0.001
	2	32 (54.2)	22 (37.3)		11 (18.6)	
	3	0 (0.0)	37 (62.7)		19 (32.2)	
	4	0 (0.0)	0 (0.0)		22 (37.3)	
	5	0 (0.0)	0 (0.0)		7 (11.9)	
4. Urinary incontinence is “still in my head.”	1	19 (32.2)	0 (0.0)	<0.001	0 (0.0)	<0.001
	2	32 (54.2)	21 (35.6)		2 (3.4)	
	3	8 (13.6)	38 (64.4)		25 (42.4)	
	4	0 (0.0)	0 (0.0)		24 (40.7)	
	5	0 (0.0)	0 (0.0)		8 (13.6)	
5. Urinary incontinence makes me feel sick.	1	23 (39.0)	0 (0.0)	<0.001	0 (0.0)	<0.001
	2	30 (50.8)	17 (28.8)		4 (6.8)	
	3	6 (10.2)	34 (57.6)		22 (37.3)	
	4	0 (0.0)	8 (13.6)		26 (44.1)	
	5	0 (0.0)	0 (0.0)		7 (11.9)	
6. I feel helpless because of urinary incontinence.	1	15 (25.4)	0 (0.0)	<0.001	0 (0.0)	<0.001
	2	31 (52.5)	24 (40.7)		10 (16.9)	
	3	13 (22.0)	35 (59.3)		18 (30.5)	
	4	0 (0.0)	0 (0.0)		24 (40.7)	
	5	0 (0.0)	0 (0.0)		7 (11.9)	
7. Urinary incontinence reduces the feeling of joy of life.	1	26 (44.1)	0 (0.0)	<0.001	0 (0.0)	<0.001
	2	33 (55.9)	11 (18.6)		4 (6.8)	
	3	0 (0.0)	35 (59.3)		22 (37.3)	
	4	0 (0.0)	13 (22.0)		26 (44.1)	
	5	0 (0.0)	0 (0.0)		7 (11.9)	
8. Urinary incontinence limits my possibilities of choosing clothes.	1	27 (45.8)	0 (0.0)	<0.001	0 (0.0)	<0.001
	2	31 (52.5)	11 (18.6)		1 (1.7)	
	3	1 (1.7)	39 (66.1)		25 (42.4)	
	4	0 (0.0)	9 (15.3)		25 (42.4)	
	5	0 (0.0)	0 (0.0)		8 (13.6)	
9. I’m afraid of having intercourse because of urinary incontinence.	1	26 (44.1)	0 (0.0)	<0.001	0 (0.0)	<0.001
	2	33 (55.9)	8 (13.6)		4 (6.8)	
	3	0 (0.0)	26 (44.1)		22 (37.3)	
	4	0 (0.0)	20 (33.9)		26 (44.1)	
	5	0 (0.0)	5 (8.5)		7 (11.9)	

Table 6 shows the distribution according to social embarrassment responses to the I-QOL questionnaire preoperatively and at six and 12 months postoperatively.

**Table 6:** Distribution according to social embarrassment responses to I-QOL questionnaire preoperatively and at six and 12 months postoperatively (N=59) Values are presented as frequency (%). ^a^p-value was calculated using the Chi-square test between observations taken preoperatively and at six months postoperatively. ^b^p-value was calculated using the Chi-square test between observations taken preoperatively and at 12 months postoperatively. I-QOL: Incontinence Quality of Life

Items	Response	Preoperatively	At 6 months postoperatively	p-value^a^	At 12 months postoperatively	p-value^b^
1. I’m afraid that people around me smell urine.	1	25 (42.4)	0 (0.0)	<0.001	0 (0.0)	<0.001
	2	33 (55.9)	17 (28.8)		5 (8.5)	
	3	1 (1.7)	34 (57.6)		24 (40.7)	
	4	0 (0.0)	8 (13.6)		27 (45.7)	
	5	0 (0.0)	0 (0.0)		3 (5.1)	
2. I’m afraid that this problem will increase with age.	1	26 (44.1)	0 (0.0)	<0.001	0 (0.0)	<0.001
	2	31 (52.5)	18 (30.5)		3 (5.1)	
	3	2 (3.4)	38 (64.4)		22 (37.3)	
	4	0 (0.0)	3 (5.1)		24 (40.7)	
	5	0 (0.0)	0 (0.0)		10 (16.9)	
3. I’m afraid of being humiliated because of urinary incontinence.	1	28 (47.5)	0 (0.0)	<0.001	0 (0.0)	<0.001
	2	31 (52.5)	11 (18.6)		3 (5.1)	
	3	0 (0.0)	37 (62.7)		20 (33.9)	
	4	0 (0.0)	11 (18.6)		25 (42.4)	
	5	0 (0.0)	0 (0.0)		11 (18.6)	
4. I’m afraid of uncontrolled urine leakage.	1	18 (30.5)	0 (0.0)	<0.001	0 (0.0)	<0.001
	2	32 (54.2)	11 (18.6)		4 (6.8)	
	3	9 (15.3)	37 (62.7)		23 (38.9)	
	4	0 (0.0)	11 (18.6)		24 (40.7)	
	5	0 (0.0)	0 (0.0)		8 (13.6)	
5. I feel like I can’t control my bladder.	1	25 (42.4)	0 (0.0)	<0.001	0 (0.0)	<0.001
	2	33 (55.9)	17 (28.8)		3 (5.1)	
	3	1 (1.7)	34 (57.6)		22 (37.3)	
	4	0 (0.0)	8 (13.6)		24 (40.7)	
	5	0 (0.0)	0 (0.0)		10 (16.9)	

## References

[1] Abrams P, Cardozo L, Fall M (2003). Standardisation Sub-committee of the International Continence Society. The standardisation of terminology in lower urinary tract function: report from the Standardisation Sub-committee of the International Continence Society. Urol.

[2] Haylen BT, de Ridder D, Freeman RM (2010). An International Urogynecological Association (IUGA)/International Continence Society (ICS) joint report on the terminology for female pelvic floor dysfunction. Neurourol Urodyn.

[3] Witkoś J, Wróbel P, Błońska-Fajfrowska B (2017). Stress urinary incontinence in women as a medical, social, psychological and economic problem - assessing the extent of knowledge of students graduating in medical fields. Med Rehabil.

[4] Ptak M, Brodowska A, Ciećwież S, Rotter I (2017). Quality of life in women with stage 1 stress urinary incontinence after application of conservative treatment-a randomized trial. Int J Environ Res Public Health.

[5] Moroni RM, Magnani PS, Haddad JM, Castro Rde A, Brito LG (2016). Conservative treatment of stress urinary incontinence: a systematic review with meta-analysis of randomized controlled trials. Rev Bras Ginecol Obstet.

[6] Magon N, Kalra B, Malik S, Chauhan M (2011). Stress urinary incontinence: what, when, why, and then what?. J Midlife Health.

[7] Luber KM (2004). The definition, prevalence, and risk factors for stress urinary incontinence. Rev Urol.

[8] Chow PM, Chuang YC, Hsu KC, Shen YC, Liu SP (2022). Impact of female stress urinary incontinence on quality of life, mental health, work limitation, and healthcare seeking in China, Taiwan, and South Korea (LUTS Asia): results from a cross-sectional, population-based study. Int J Womens Health.

[9] Huang ZM, Xiao H, Ji ZG, Yan WG, Zhang YS (2018). TVT versus TOT in the treatment of female stress urinary incontinence: a systematic review and meta-analysis. Ther Clin Risk Manag.

[10] Kołodyńska G, Zalewski M, Fink-Lwow F, Mucha A, Andrzejewski W (2021). Quality of life of physically active and inactive women who are older after surgery for stress urinary incontinence using a transobturator tape (TOT). J Clin Med.

[11] Zalewski M, Kołodyńska G, Mucha A, Andrzejewski W (2021). A prospective study of the quality of life of patients with stress incontinence before and after a transobturator tape (TOT) procedure-preliminary report. J Clin Med.

[12] Jacob KJ, Jayaprakash M, Cherian A (2017). Quality of life assessment in women with stress urinary incontinence following Trans Obturator Tape (TOT) insertion: a prospective study. Int J Reprod Contracept Obstet Gynecol.

[13] Ajith AK, Rekha A, Duttagupta S, Murali V, Ramakrishnan D, Krishnapillai V (2019). Prevalence and factors of urinary incontinence among postmenopausal women attending the obstetrics and gynecology outpatient service in a tertiary health care center in Kochi, Kerala. Indian J Community Med.

[14] Ferreira CH, Bø K (2015). The pad test for urinary incontinence in women. J Physiother.

[15] Chang YW, Chuang FC, Wu LY, Yang TH, Kung FT, Huang KH (2019). Evaluating the efficacy of the single-incision uphold system for pelvic organ prolapse repair. Taiwan J Obstet Gynecol.

[16] Lin L, Huang MC, Su TH, Lau HH (2018). Comparison between tension-free vaginal tape and transobturator tape in treating stress urinary incontinence after vaginal mesh surgery. Taiwan J Obstet Gynecol.

[17] Chen G, Tan JT, Ng K, Iezzi A, Richardson J (2014). Mapping of incontinence quality of life (I-QOL) scores to assessment of quality of life 8D (AQoL-8D) utilities in patients with idiopathic overactive bladder. Health Qual Life Outcomes.

[18] Nojomi M, Baharvand P, Moradi Lakeh M, Patrick DL (2009). Incontinence quality of life questionnaire (I-QOL): translation and validation study of the Iranian version. Int Urogynecol J Pelvic Floor Dysfunct.

[19] Orhan C, Ozgul S, Baran E (2020). The effect of incontinence severity on symptom distress, quality of life, and pelvic floor muscle function in Turkish women with urinary incontinence. Gynecol Obstet Reprod Med.

[20] Bushnell DM, Martin ML, Summers KH, Svihra J, Lionis C, Patrick DL (2005). Quality of life of women with urinary incontinence: cross-cultural performance of 15 language versions of the I-QOL. Qual Life Res.

[21] Mallah F, Montazeri A, Ghanbari Z, Tavoli A, Haghollahi F, Aziminekoo E (2014). Effect of urinary incontinence on quality of life among Iranian women. J Family Reprod Health.

[22] Goldacre MJ, Abisgold JD, Yeates DG, Voss S, Seagroatt V (2007). Self-harm and depression in women with urinary incontinence: a record-linkage study. BJU Int.

[23] Irwin DE, Milsom I, Kopp Z, Abrams P, Cardozo L (2006). Impact of overactive bladder symptoms on employment, social interactions and emotional well-being in six European countries. BJU Int.

